# Alteration of ROS Homeostasis and Decreased Lifespan in *S. cerevisiae* Elicited by Deletion of the Mitochondrial Translocator *FLX1*

**DOI:** 10.1155/2014/101286

**Published:** 2014-05-08

**Authors:** Teresa Anna Giancaspero, Emilia Dipalo, Angelica Miccolis, Eckhard Boles, Michele Caselle, Maria Barile

**Affiliations:** ^1^Istituto di Biomembrane e Bioenergetica, CNR, Via Amendola 165/A, 70126 Bari, Italy; ^2^Dipartimento di Bioscienze, Biotecnologie e Biofarmaceutica, Università degli Studi di Bari “A. Moro”, Via Orabona 4, 70126 Bari, Italy; ^3^Institute of Molecular Biosciences, Goethe University Frankfurt, Max-von-Laue Straße 9, 60438 Frankfurt am Main, Germany; ^4^Dipartimento di Fisica, Via P. Giuria 1, 10125 Torino, Italy

## Abstract

This paper deals with the control exerted by the mitochondrial translocator *FLX1*, which catalyzes the movement of the redox cofactor FAD across the mitochondrial membrane, on the efficiency of ATP production, ROS homeostasis, and lifespan of *S. cerevisiae*. The deletion of the *FLX1* gene resulted in respiration-deficient and small-colony phenotype accompanied by a significant ATP shortage and ROS unbalance in glycerol-grown cells. Moreover, the *flx*1Δ strain showed H_2_O_2_ hypersensitivity and decreased lifespan. The impaired biochemical phenotype found in the *flx*1Δ strain might be justified by an altered expression of the flavoprotein subunit of succinate dehydrogenase, a key enzyme in bioenergetics and cell regulation. A search for possible *cis*-acting consensus motifs in the regulatory region upstream SDH1-ORF revealed a dozen of upstream motifs that might respond to induced metabolic changes by altering the expression of Flx1p. Among these motifs, two are present in the regulatory region of genes encoding proteins involved in flavin homeostasis. This is the first evidence that the mitochondrial flavin cofactor status is involved in controlling the lifespan of yeasts, maybe by changing the cellular succinate level. This is not the only case in which the homeostasis of redox cofactors underlies complex phenotypical behaviours, as lifespan in yeasts.

## 1. Introduction

Riboflavin (Rf or vitamin B_2_) is the precursor of flavin mononucleotide (FMN) and flavin adenine dinucleotide (FAD), the redox cofactors of a large number of dehydrogenases, reductases, and oxidases. Most of these flavoenzymes are compartmented in the cellular organelles, where they are involved in energy production and redox homeostasis as well as in different cellular regulatory events including apoptosis, chromatin remodelling, and interestingly, as recently proposed, in epigenetic signalling [1–4]. Consistent with the crucial role of flavoenzymes in cell life, flavin-dependent enzyme deficiency and/or impairment in flavin homeostasis in humans and experimental animals has been linked to several diseases, such as cancer, cardiovascular diseases, anaemia, abnormal fetal development, and different neuromuscular and neurological disorders [5–9]. The relevance of these pathologies merits further research aimed to better describe FAD homeostasis and flavoenzyme biogenesis, especially in those organisms that can be a simple and suitable model for human diseases. The conserved biological processes shared with all eukaryotic cells, together with the possibility of simple and quick genetic manipulation, allowed proposing the budding yeast,* Saccharomyces cerevisiae*, as the premier model to understand the biochemistry and molecular biology of mammalian cells and to decipher molecular mechanisms underlying human diseases [10–12].

For many years* S. cerevisiae* has been used also as a model to study the complexity of the molecular events involved in the undesired process of aging, in which mitochondria play a major role [13, 14]. The role of mitochondria has been pointed out either because aged respiratory chain is a major source of cellular ROS [14] or because mitochondria actively participate in regulating the homeostasis of the redox cofactor NAD, which regulates yeast lifespan by acting as a substrate of specific deacetylases (EC 3.5.1.-), named sirtuins [15–17]. This might not be the only case in which the homeostasis of redox cofactors underlies complex phenotypical behaviours, as lifespan in yeasts. Here we investigate whether the mitochondrial flavin cofactor status may also be involved in controlling the lifespan of yeasts, presumably by changing the level of mitochondrial flavoenzymes, which are crucial for cell regulation [18, 19].

It should be noted that, even though mitochondria are plenty of flavin and flavoproteins [20, 21], the origin of flavin cofactors starting from Rf in this organelle is still a matter of debate. Yeasts have the ability to either synthesise Rf* de novo* or to take it from outside. The first eukaryotic gene coding for a cellular Rf transporter was identified in* S. cerevisiae* as the* MCH5* gene [22]. Intracellular Rf conversion to FAD is a ubiquitous pathway and occurs via the sequential actions of ATP: riboflavin 5′-phosphotransferase or riboflavin kinase (RFK, EC 2.7.1.26) which phosphorylates the vitamin into FMN and of ATP: FMN adenylyl transferase or FAD synthase (FADS, EC 2.7.7.2) which adenylates FMN to FAD. The first eukaryotic genes encoding for RFK and FADS were identified in* S. cerevisiae* and named* FMN1* [23] and* FAD1* [24], respectively. While there is no doubt about a mitochondrial localization for Fmn1p [23, 25], the existence of a mitochondrial FADS isoform in yeast is still controversial. First a cytosolic localization for Fad1p was reported [24]; thus newly synthesised FAD was expected to be imported into mitochondria via the FAD translocator Flx1p [25]. However, results from our laboratory showed that, besides in the cytosol, FAD-forming activities can be revealed in mitochondria, thus requiring uptake of the FAD precursors into mitochondria [26, 27]. FAD synthesised inside the organelle can be either delivered to a number of nascent client apo-flavoenzymes or be exported via Flx1p into cytosol to take part of an extramitochondrial posttranscriptional control of apo-flavoprotein biogenesis [19, 26].

Besides synthesis and transport, mitochondrial flavin homeostasis strictly depends also on flavin degradation. Recently we have demonstrated that* S. cerevisiae* mitochondria (SCM) are able to catalyze FAD hydrolysis via an enzymatic activity which is different from the already characterized NUDIX hydrolases (i.e., enzymes that catalyze the hydrolysis of nucleoside diphosphates linked to other moieties, X) and it is regulated by the mitochondrial NAD redox status [17].

To prove the relationship between mitochondrial FAD homeostasis and lifespan in yeast we use as a model a* S. cerevisiae* strain lacking the* FLX1* gene which showed a respiratory-deficient phenotype and a derangement in a number of mitochondrial flavoproteins, that is, dihydrolipoamide dehydrogenase (*LPD1*), succinate dehydrogenase (*SDH*), and flavoproteins, involved in ubiquinone biosynthesis (*COQ6*) [18, 25, 26, 28].

We demonstrated here that this deleted strain performed ATP shortage and ROS unbalance, together with H_2_O_2_ hypersensitivity and altered chronological lifespan. This* flx1Δ* phenotype is correlated to a reduced ability to maintain an appropriate level of the flavoenzyme succinate dehydrogenase (SDH), a member of a complex “flavin network” participating in a nucleus-mitochondrion cross-talk.

## 2. Materials and Methods

### 2.1. Materials

All reagents and enzymes were from Sigma-Aldrich (St. Louis, MO, USA). Zymolyase was from ICN (Abingdon, UK) and Bacto Yeast Extract and Bacto Peptone were from Difco (Franklin Lakes, NJ, USA). Mitochondrial substrates were used as TRIS salts at pH 7.0. Solvents and salts used for HPLC were from J. T. Baker (Center Valley, PA, USA). Rat anti-HA monoclonal antibody and peroxidase-conjugated anti-rat IgG secondary antibody were obtained from Roche (Basel, Switzerland) and Jackson Immunoresearch (West Grove, PA, USA), respectively.

### 2.2. Yeast Strains

The wild-type* S. cerevisiae* strain (EBY157A or* WT* genotype* MAT*α* ura 3–52 MAL2-8* ^*c*^ *SUC2 p426MET25*) used in this work derived from the CEN.PK series of yeast strains and was obtained from P. Kotter (Institut für Mikrobiologie, Goethe-Universität Frankfurt, Frankfurt, Germany), as already described in [26]. The* flx1Δ* mutant strain (EBY167A,* flx1Δ*) was constructed as described in [26] and the* WT-HA* (EBY157-SDH1-HA) and* flx1Δ-HA* (EBY167-G418S-SDH1-HA) were constructed as described in [19].

### 2.3. Media and Growth Conditions

Cells were grown aerobically at 30°C with constant shaking in rich liquid medium (YEP, 10 g/L Yeast Extract, 20 g/L Bacto Peptone) or in minimal synthetic liquid medium (SM, 1.7 g/L yeast nitrogen base, 5 g/L ammonium sulphate, and 20 mg/L uracil) supplemented with glucose or glycerol (2% each) as carbon sources. The YEP or SM solid media contained 18 g/L agar.

### 2.4. Chronological Lifespan Determination

WT and* flx1Δ* strains were grown overnight at 30°C in 5 mL YEP liquid medium supplemented with glucose 0.5% up to the early stationary phase. Each strain was then cultured in SM liquid medium at 30°C for 1, 4, and 7 days. Five serial dilutions from each culture containing 200 cells, calculated from A_600 nm_, were plated onto SM solid medium and grown at 30°C for two-three days.

### 2.5. H_2_O_2_ Sensitivity

*WT* and* flx1Δ* strains were grown overnight at 30°C in 5 mL YEP liquid medium supplemented with glucose 0.5% up to the early stationary phase. Then, each strain was inoculated in SM liquid medium (initial A_600 nm_ equal to 0.1) containing glucose 2% and H_2_O_2_ (0.05 or 2 mM). After 5 or 24 h of growth at 30°C, the H_2_O_2_ sensitivity was estimated by measuring the A_600 nm_ of the growth culture.

### 2.6. Malate and Succinate Sensitivity

*WT* and* flx1Δ* strains were grown overnight at 30°C in 5 mL YEP liquid medium supplemented with glucose 0.5% up to the early stationary phase. Then, each strain was inoculated in SM liquid medium (initial A_600 nm_ equal to 0.1) containing glucose 2% and succinate or malate (5 mM). After 24 h of growth at 30°C, the H_2_O_2_ sensitivity was estimated by measuring the A_600 nm_ of the growth culture.

### 2.7. Preparation of Spheroplasts, Mitochondria, and Cellular Lysates

Spheroplasts were prepared using Zymolyase. Mitochondria were isolated from spheroplasts as described in [26]. Cellular lysates were obtained by early exponential-phase (5 h) or stationary-phase (24 h) cells harvested by centrifugation (8000 ×g for 5 min), washed with sterile water, resuspended in 250 *μ*L of lysis buffer (10 mM Tris-HCl, pH 7.6, 1 mM EDTA, 1 mM dithiothreitol, and 0.2 mM phenylmethanesulfonyl fluoride, supplemented with one tablet of Roche protease inhibitor cocktail every 10 mL of lysis buffer), and vortexed with glass beads for 10 min at 4°C. The liquid was removed and centrifuged at 3000 ×g for 5 min to remove cell debris. The protein concentrations of the spheroplasts, mitochondria, and cellular lysates were assayed according to Bradford [29].

### 2.8. Quantitation of Flavins, ATP, and Reactive Oxygen Species (ROS)

Rf, FMN, and FAD content in spheroplasts and SCM was measured in aliquots (5–80 *μ*L) of neutralized perchloric extracts by means of HPLC (Gilson HPLC system including a model 306 pump and a model 307 pump equipped with a Kontron Instruments SFM 25 fluorometer and Unipoint system software), essentially as previously described [26]. ATP content was measured fluorometrically in cellular lysates by using the ATP Detecting System, essentially as in [30]. NADPH formation, which corresponds to ATP content (with a 1 : 1 stoichiometry), was followed with excitation wavelength at 340 nm and emission wavelength at 456 nm. ROS level was fluorometrically measured on cellular lysates using as substrate 2′-7′-dichlorofluorescin diacetate (DCF-DA) according to [30], with slight modifications. Briefly, the probe DCF-DA (50 *μ*M) was incubated at 37°C for 1 h with 0.03–0.05 mg proteins and converted to fluorescent dichlorofluorescein (DCF) upon reaction with ROS. DCF fluorescence of each sample was measured by means of a LS50S Perkin Elmer spectrofluorometer (excitation and emission wavelengths set at 485 nm and 520 nm, resp.).

### 2.9. Enzymatic Assays

Succinate dehydrogenase (SDH, EC 1.3.5.1) and fumarase (FUM, EC 4.2.1.2) activities were measured as in [26]. Glutathione reductase (GR, EC 1.6.4.2) activity was spectrophotometrically assayed by monitoring the absorbance at 340 nm due to NADPH oxidation after glutathione addition (1 mM), essentially as in [30]. Superoxide dismutase (SOD, EC 1.15.1.1) activity was spectrophotometrically measured by the xanthine oxidase/xanthine/cytochrome c method, essentially as described in [31].

### 2.10. Statistical Analysis

All experiments were repeated at least three times with different cell preparations. Results are presented as mean ± standard deviation (SD). Statistical significance was evaluated by Student's *t*-test. Values of *P* < 0.05 were considered statistically significant.

## 3. Results

### 3.1. Phenotypical and Biochemical Consequences of* FLX1* Deletion

**Figure 1:**
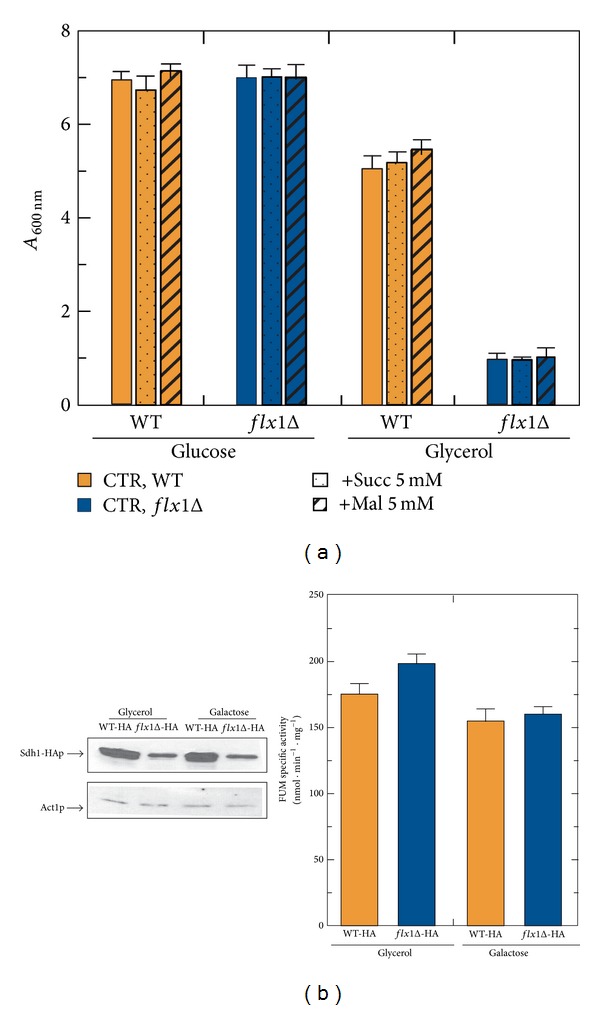
(a) Respiratory-deficient phenotype of* flx1Δ* strain: effect of succinate and malate addition. WT and* flX1Δ* cells were cultured at 30°C in YEP liquid medium supplemented with either glucose or glycerol (2% each) as carbon source. Where indicated either 5 mM succinate (Succ) or 5 mM malate (Mal) was added. Cell growth was estimated at the stationary phase (24 h) by measuring the absorbance at 600 nm (*A*_600 nm_) of a ten-fold dilution of each growth culture, consistently, corrected for the dilution factor. The values reported in the histogram are the means (±SD) of three experiments. (b) Changes in the recombinant Sdh1-HAp level in* flx1Δ* strain. Cellular lysates were prepared from* WT-HA* and* flX1Δ-HA* cells grown at 30°C up to the exponential growth phase (5 h) in YEP liquid medium supplemented with either glycerol or galactose (2% each) as carbon source. Proteins from cellular lysates (0.05 mg) were separated by SDS/PAGE and transferred onto a PVDF membrane. In each extract, Sdh1-HA protein was detected by using an *α*-HA and its amount was densitometrically evaluated. The values reported in the histogram are the means (±SD) of three experiments performed with different cellular lysates preparations. Statistical evaluation was carried out according to Student's *t*-test (*P < 0.05). As a control, the specific activity of the enzyme fumarase (FUM) was determined in each cellular lysate preparation.

In order to study the relevance of mitochondrial flavin cofactor homeostasis on cellular bioenergetics we introduced a yeast strain lacking the* FLX1* gene, encoding the mitochondrial FAD transporter [26]. This deleted strain showed a small-colony phenotype, on both fermentable and nonfermentable carbon sources, due to an impairment in the aerobic respiratory chain pathway [32]. The deleted strain,* flx1Δ*, grew normally on glucose medium but failed to grow on nonfermentable carbon sources (i.e., glycerol), thus indicating a respiration-deficient phenotype (Figure 1(a)). The growth defect on nonfermentable carbon source, which was restored by complementing the deleted strain with the YEpFLX1 plasmid [26], was not rescued by the addition of tricarboxylic acid (TCA) cycle intermediates such as succinate or malate (Figure 1(a)).

Among the mitochondrial flavoenzymes which were demonstrated to be altered in* flx1Δ* strain [25, 26, 28], we showed before [19, 32] and confirmed in Figure 1(b) a significant reduced level of the apo-flavoprotein Sdh1p, resulting in an altered functionality of SDH or complex II of the respiratory chain. This reduction was revealed by creating a strain in which three consecutive copies of the human influenza hemagglutinin epitope (HA epitope, YPYDVPDYA) were fused in frame to the 3′end of the* SDH1 ORF* in the genome of both the WT and* flx1Δ* strains. The chimera protein, namely, Sdh1-HAp, carrying the HA-tag at the C-terminal end of Sdh1p, lost the ability to covalently bind the flavin cofactor FAD [19, 33], but not its regulatory behaviour, that is, its inducible expression in galactose or in nonfermentable carbon sources. In all the growth conditions tested, the FAD-independent fumarase (FUM) activity, used as a control, was not affected by* FLX1* deletion (see histogram in Figure 1(b)).

A significant decrease of Sdh1-HAp level was accompanied in galactose, but not in glycerol, by a profound derangement of flavin cofactors, particularly evident in cell grown at the early exponential phase (Table 1), in agreement with [25, 26], respectively. The reason for these carbon source-dependent flavin level changes, which is not easily explainable, is addressed in Section 4.

**Figure 2:**
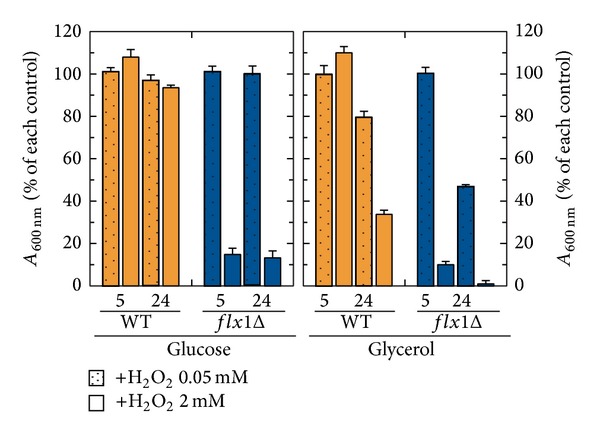
Sensitivity to H_2_O_2_. WT and* flX1Δ* cells were cultured at 30°C in YEP liquid medium supplemented with either glucose or glycerol (2% each) as carbon source. Where indicated, H_2_O_2_ at the indicated concentration was added. Cell growth was estimated at the exponential (5 h) and stationary phase (24 h) by measuring the absorbance at 600 nm (*A*_600 nm_). In the histogram, the *A*_600 nm_ of the cell cultures grown in the presence of H_2_O_2_ is reported as a percentage of the control (i.e., the *A*_600 nm_ of cell cultures grown in the absence of H_2_O_2_, set arbitrary equal to 100%). The values reported in the histogram are the means (±SD) of three experiments.

Consistent with an altered functionality of SDH, the* flx1Δ* strain also showed impaired isolated mitochondria oxygen consumption activity, specifically detectable when succinate was used as a respiratory substrate [19]. Similar phenotype was also observed in yeast strains carrying either a deletion of* SDH1* [34] or a deletion of* SDH5*, which encodes a mitochondrial protein involved in Sdh1p flavinylation [35]. Another respiration-related phenotype of* flx1Δ* strain was investigated in Figure 2, by testing H_2_O_2_ hypersensitivity of cells grown on both fermentable and nonfermentable carbon sources. In glucose, the WT cells grew up to the stationary phase (24 h) in the presence of H_2_O_2_ (0.05 or 2 mM) essentially as the control cells grown in the absence of H_2_O_2_. In glycerol, their ability to grow up to 24 h was reduced of about 20% at 0.05 mM H_2_O_2_ and of 60% at 2 mM, with respect to the control cells in which no H_2_O_2_ was added.

In glucose,* flx1Δ* cells did not show H_2_O_2_ hypersensitivity at 0.05 mM. At 2 mM H_2_O_2_, their ability to grow was significantly reduced (of about 85%) with respect to* flx1Δ* cells grown in the absence of H_2_O_2_. The ability of the* flx1Δ* cells to grow in glycerol, which was* per se* drastically reduced by deletion, was reduced at 24 h by the addition of 0.05 mM H_2_O_2_ (about 50% with respect to the control cells grown in the absence of H_2_O_2_). An even higher sensitivity to H_2_O_2_ was observed in the presence of 2 mM H_2_O_2_, having their growth ability reduced of about 85% with respect to control cells in which no addition was made. The impairment in the ability to grow under H_2_O_2_ stress conditions clearly demonstrates an impairment in defence capability of the* flx1Δ* strain. Interestingly, the same phenotype was observed also in the yeast* sdh5Δ* [35],* sdh1Δ*, and* sdh2Δ* [36] strains.

**Figure 3:**
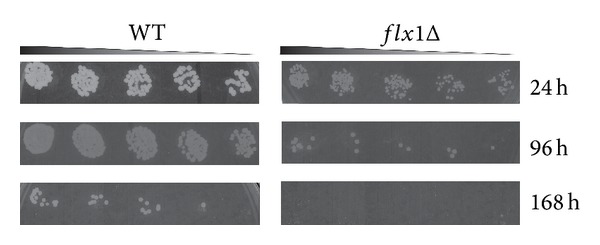
Chronological lifespan determination. WT and* flX1Δ* strains were cultured in SM liquid medium at 30°C. Dilutions from each culture containing about 200 cells (as calculated from *A*_600 nm_ by taking into account that one *A*_600 nm_ is equivalent to 3 × 10^7^ cell/mL) were harvested after 24, 96, and 168 h and plated onto SM solid medium and grown at 30°C for two-three days.

To understand whether mitochondrial flavoprotein impairment, due to* FLX1* deletion, influenced aging in yeast, we carried out measurements of chronological lifespan on both WT and* flx1Δ* cells cultured at 30°C in SM liquid medium supplemented with glucose 2% as carbon source (Figure 3). Following 24 h (1 day), 96 h (4 days), and 168 h (7 days) of growth, the number of colonies was determined by spotting five serial dilutions of the liquid culture and incubating the plates for two-three days at 30°C. The results of a typical experiment are reported in Figure 3. A reduced number of small colonies were counted for the* flx1Δ* strain, with respect to the number of colonies counted for the WT strain. This phenotype, particularly evident after 96 h and 168 h of growth time, clearly indicated a decrease in chronological lifespan of the* flx1Δ* strain. Essentially the same phenotype was observed in* sdh1Δ* and* sdh5Δ* strains [35]. Thus, it seems quite clear that a correct biogenesis of mitochondrial flavoproteome, and in particular assembly of SDH, ensures a correct aging rate in yeast. When* flx1Δ cells* were grown on glycerol, they lost the ability to form colonies following 24 h growth time (data not shown).

**Figure 4:**
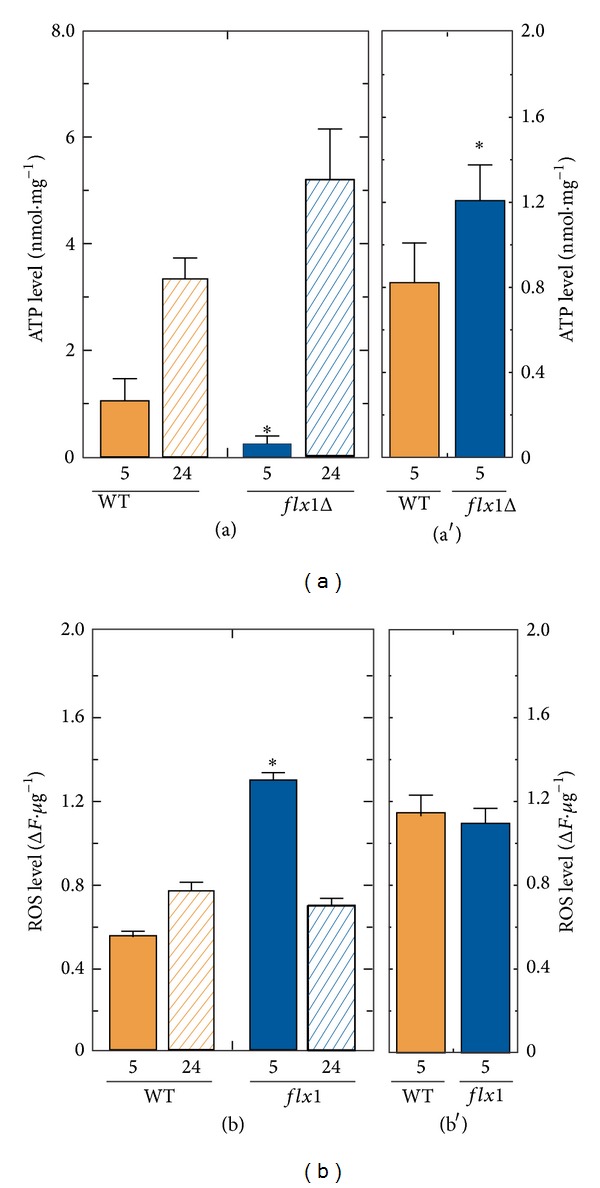
Bioenergetic and redox impairment in* flx1Δ* strain: ATP and ROS content. Cellular lysates were prepared from WT and* flx1Δ* mutant strains grown in glycerol ((a), (b)) up to either the exponential (5 h) or the stationary phase (24 h) or in glucose ((a′), (b′)) up to the exponential phase (5 h). ATP content ((a), (a′)) was enzymatically determined following perchloric acid extraction and neutralization. ROS content ((b), (b′)) was fluorometrically measured as described in Section 2. The values reported in the histograms are the means (±SD) of three experiments performed with different cellular lysate preparations. Statistical evaluation was carried out according to Student's *t*-test (*P < 0.05).

In order to correlate the observed phenotype with an impairment of cellular bioenergetics, we compared the ATP content and the ROS amount of the* flx1Δ* strain with that of the WT. In Figure 4, panel (a), the ATP cellular content was enzymatically measured in neutralized perchloric extracts prepared from WT and* flx1Δ* cells grown on glycerol. At the exponential growth phase (5 h), a significant reduction was detected in the* flx1Δ* cells in comparison with the WT (0.21 versus 1.05 nmol·mg^−1^ protein). At the stationary growth phase (24 h), the ATP content increased significantly in WT cells (3.4 nmol·mg^−1^ protein) and even more in the deleted strain (5.2 nmol·mg^−1^ protein). The temporary severe decrease in ATP content induced by the absence of Flx1p was not observed in glucose-grown cells (Figure 4, panel (a′)), as expected when fermentation is the main way to produce ATP.

*FLX1* deletion induced also a significant increase in the amount of ROS (135% with respect to the WT cells), as estimated with the fluorescent dye DCFH-DA on the cellular lysates prepared from cells grown in glycerol up to the exponential growth phase (Figure 4, panel (b)). At the stationary phase the* flx1Δ* cells presented almost the same ROS amount measured in the WT cells (Figure 4, panel (b)). In glucose-grown cells, the amount of cellular ROS in the* flx1Δ* strain was not significantly changed with respect to the WT (Figure 4, Panel (b′)), as expected when a mitochondrial damage is the major cause of ROS unbalance.

**Figure 5:**
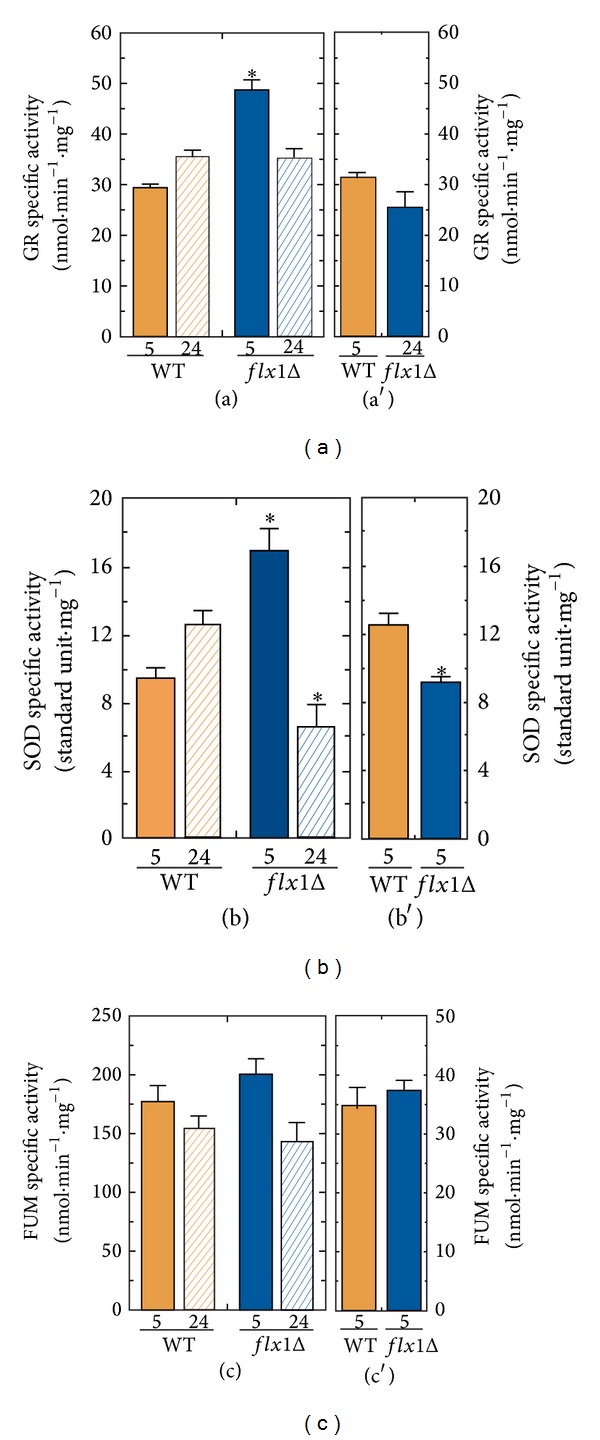
GR and SOD activities in* flx1Δ* strain. Cellular lysates were prepared from WT and* flx1Δ* mutant strains grown in glycerol ((a), (b), and (c)) up to either the exponential (5 h) or the stationary phase (24 h) or in glucose ((a′), (b′), and (c′)) up to the exponential phase (5 h). GR ((a), (a′)) and SOD ((b), (b′)) specific activities were spectrophotometrically determined as described in Section 2. As control FUM specific activity ((c), (c′)) was measured as described in Section 2. The values reported in the histograms are the means (±SD) of three experiments performed with different cellular lysate preparations. Statistical evaluation was carried out according to Student's *t*-test (*P < 0.05).

In line with the unique role of flavin cofactor in oxygen metabolism and ROS defence systems [20, 30, 37, 38], we further investigated whether the impairment of the ROS level in glycerol-grown* flx1Δ* strain was due to a derangement in enzymes involved in ROS detoxification, such as the flavoprotein glutathione reductase (GR) or the FAD-independent superoxide dismutase (SOD); their specific enzymatic activities were measured in cellular lysates from WT and* flx1Δ* cells grown on glycerol and glucose, while assaying the FAD-independent enzyme FUM as control (Figure 5). Figure 5, panel (a), shows a significant increase in GR specific activity in* flx1Δ* strain (65%) at the exponential growth phase with respect to that measured in WT. The GR specific activity in the* flx1Δ* reached the same value measured in the WT cells (about 35 nmol·mg^−1^ protein) at the stationary phase. In cells grown in glucose up to the exponential growth phase (Figure 5, panel (a′)) a slight, but not significant, reduction in GR specific activity was detected in the* flx1Δ* strain with respect to the WT (25 versus 31 nmol·mg^−1^ protein).

As regards SOD, in the glycerol-grown* flx1Δ* cells after 5 h growth time (Figure 5, panel (b)), the SOD specific activity was significantly higher than the value measured in the WT cells (16 versus 9 standard U·mg^−1^). At the stationary phase, the SOD specific activity in the* flx1Δ* significantly decreased, reaching a value of 6.6 standard U·mg^−1^, that is, about two-fold lower than the SOD specific activity measured in WT cells. In glucose-grown cells after 5 h growth time (Figure 5, panel (b′)), a slight, but significant, reduction in SOD specific activity can be detected in the* flx1Δ* strain with respect to the WT (9.2 versus 12.2 nmol·mg^−1^ protein). This reduction might be explained by a defect in FAD dependent protein folding, as previously observed in [30, 39].

In all the growth conditions tested, the FUM activity, used as a control, was not affected by* FLX1 *deletion (Figure 5, panels (c) and (c′)).

### 3.2. The Role of Flx1p in a Retrograde Cross-Talk Response Regulating Cell Defence and Lifespan

Results described in the previous paragraph strengthen the relevance of Flx1p in ensuring cell defence and correct aging by maintaining the homeostasis of mitochondrial flavoproteome. As concerns SDH, in [19] we gained some insight into the mechanism by which Flx1p could regulate Sdh1p apo-protein expression, as due to a control that involves regulatory sequences located upstream of the SDH1 coding sequence (as reviewed in [40]).

To gain further insight into this mechanism, we searched here for elements that could be relevant in modulating Sdh1p expression, in response to alteration in flavin cofactor homeostasis. Therefore, first we searched for* cis*-acting elements in the regulatory regions located upstream of the* SDH1 ORF*, first of all in the 5′UTR region, as defined by [41], which corresponds to the first 71 nucleotides before the start codon of* SDH1 ORF*. No consensus motifs were found in this region by using the bioinformatic tool “Yeast Comparative Genomics—Broad Institute” [42]. Indeed, it should be noted that no further information is at the moment available on the actual length of the 5′UTR of* SDH1*.

Thus, we extended our analysis along the 1 kbp upstream region of* SDH1 ORF* and we found twelve consensus motifs that could bind regulatory proteins, six of which are of unknown function. Among these motifs, summarised in Table 2, the most relevant, at least in the scenario described by our experiments, seemed to be a motif which is located at −80 nucleotides upstream the start codon of* SDH1 ORF* and, namely, motif 29 (consensus sequence shRCCCYTWDt), that perfectly overlaps with motif 38 (consensus sequence CTCCCCTTAT). This motif is also present in the upstream region of the mitochondrial flavoprotein* ARH1*, involved in ubiquinone biosynthesis [28], but not in that of flavoprotein* LPD1* and* COQ6* [25, 26, 28]. Interestingly, this motif 29 is also present in the upstream regions of the members of the machinery that maintained Rf homeostasis, that is, the mitochondrial FAD transporter* FLX1* [25], the FAD forming enzyme* FAD1* [25], and the Rf translocator* MCH5* [22]. Moreover, this motif is also present in the upstream regulatory region of the mitochondrial isoenzyme* SOD2*, but not in the cytosolic one,* SOD1*, and in one of the five nuclear succinate sensitive JmjC-domain-containing demethylases, that is,* RPH1* [43]. According to [42], this motif is bound by transcription factor Msn2p and its close homologue Msn4p (referred to as Msn2/4p), which under nonstress conditions are located in the cytoplasm. Upon different stress conditions, among which oxidative stress, Msn2/4p are hyper-phosphorylated and shuttled from the cytosol to the nucleus [44]. The pivotal role played by Msn2/4p in chronological lifespan in yeast was first discovered by [45] and recently exhaustively reviewed by [46].

A further comparison between the 5′UTRs of* SDH1* and of proteins involved in FAD homeostasis revealed another common motif of unknown function located at –257 nucleotides upstream the start codon of* SDH1 ORF*, namely, the motif 14 (consensus sequence YCTATTGTT) [42]. Besides* SDH1*, this motif is also present in the upstream region of* MCH5* and its homologue* MCH4*, in* FAD1*, and also in a number of mitochondrial flavoproteins, including* HEM14*,* NDI1*, and* NCP1*. The binding factor and the functional role of the motif 14 have not yet annotated in “Yeast Comparative Genomics—Broad Institute” (Table 2). Searching in the biological database “Biobase-Gene-regulation-Transfac” we found that this motif is reported as bound by Rox1p (YPR065W, a heme-dependent repressor of hypoxic genes—SGD information). Rox1p is involved in the regulation of the expression of proteins involved in oxygen-dependent pathways, such as respiration, heme, and sterols biosynthesis [47]. Thus,* SDH1* expression is downregulated in* rox1Δ* strain under aerobiosis [47]. This finding strengthens the well-described relationship between oxygen/heme metabolism and flavoproteins [18, 37]. A possible involvement of this transcriptional pathway in the scenario depicted by deletion of* FLX1* remains at the moment only speculative.

## 4. Discussion

This paper deals with the role exerted by the mitochondrial translocator Flx1p in the efficiency of ATP production, ROS homeostasis, H_2_O_2_ sensitivity, and chronological lifespan in* S. cerevisiae*, starting from the previous demonstrations of the derangements in specific mitochondrial flavoproteins which are crucial for mitochondrial bioenergetics, including Coq6p [28], Lpd1p, and Sdh1p [19, 25, 26]. The alteration in Sdh1p expression level in different carbon source is confirmed here (Figure 1) and it is accompanied by an alteration in flavin cofactor amount in galactose, but not in glycerol-grown cells (Table 1), in agreement with [19, 25], respectively. In the attempt to rationalize the reason for the carbon source dependence of the flavin level changes, we hypothesized different subcellular localization for Fad1p in response to carbon sources. Experiments are going on in our laboratory to evaluate this possibility.

The* flx1Δ* strain showed impaired succinate-dependent oxygen consumption [19]. Since no reduction in the oxygen consumption rate was found by using alternative substrates, such as NADH or glycerol 3-phosphate, possible defects in the ubiquinone or heme biosynthesis [28] could not be relevant for mitochondrial respiration, at least under this nonstress condition.

To evaluate the consequences of* FLX1* deletion on bioenergetics and cellular redox balance, the ATP content and ROS level (Figure 4) were compared in WT and* flx1Δ* strains, accompanied by measurements of the enzymatic activities of GR and SOD, enzymes involved in ROS detoxification (Figure 5). ATP shortage and ROS unbalance were observed in* flx1Δ *cells grown in glycerol up to the exponential growth phase, but not in cells grown in glycerol up to the stationary phase or in glucose. The findings are in agreement with the mitochondrial origin of these biochemical parameters. More importantly, the observation that lifespan was changed in glucose (not accompanied by a detectable ROS unbalance) allows us to propose that the lifespan shortage induced by the mitochondrial alteration due to absence of* FLX1* gene (correlated to flavoprotein impairment) may act also independently of ROS level increase.

The* flx1Δ* strain showed also H_2_O_2_ hypersensitivity (Figure 2). Since the same respiratory-deficient phenotype was previously observed in the yeast strain* sdh1Δ* and* sdh5Δ* strains [35], these results could be explained by the incapability of the* flx1Δ* strain to increase the amount of Sdh1p in response to oxidative stress.

In this paper, for the first time, a correlation between deletion of* FLX1* and altered chronological lifespan was reported (Figure 3). A similar phenotype was also previously demonstrated for* sdh5Δ* strains [35]. Thus, it seems quite clear that a correct biogenesis of mitochondrial flavoproteome, and in particular assembly of SDH, ensures a correct aging rate in yeast. This conclusion is also consistent with the recent observations made in another model organism, that is,* C. elegans*, in which the FAD forming enzyme FADS coded by* flad-1* gene was silenced [30, 48].

To understand the molecular mechanism by which FAD homeostasis derangement and flavoproteome level maintenance are correlated, a bioinformatic analysis was performed which revealed at least two* cis*-acting motifs which are located in the upstream region of genes encoding SDH1, other mitochondrial flavoproteins, and some members of the machinery that maintain cellular FAD homeostasis. Therefore, the analysis describes the ability of yeast cells to implement under H_2_O_2_ stress condition and aging a strategy of gene expression coordinating flavin cofactor homeostasis with the biogenesis of a number of mitochondrial flavoenzymes involved in various aspects of metabolism ranging from oxidative phosphorylation to heme and ubiquinone biosynthesis. Even though no experimental evidence still exists to test the direct involvement of these* cis*-acting motifs in flavin-dependent cell defence and chronological lifespan, their involvement in the scenario depicted by deletion of* FLX1* appeared to be a fascinating purpose to be pursued. Experiments in this direction are at the moment going on in our laboratory.

**Figure 6:**
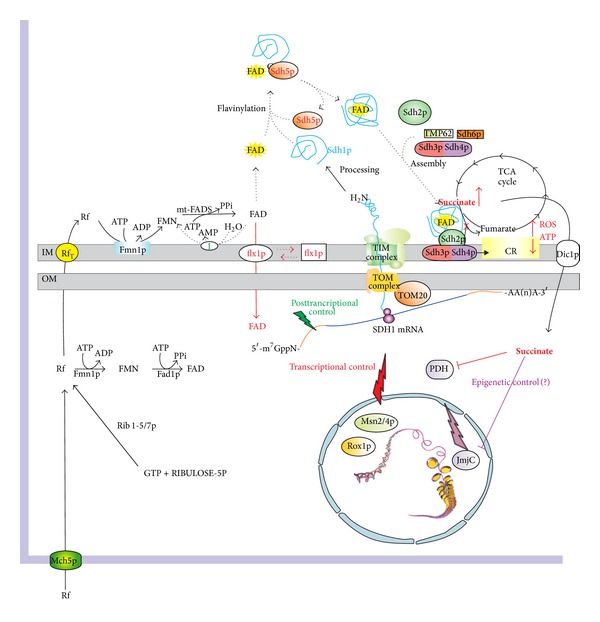
A possible correlation between mitochondrial FAD homeostasis and chronological lifespan. The scheme summarizes results from studies described in this and other papers [17, 19, 22, 26, 35, 36, 40, 50, 53]. Mch5p, plasma membrane Rf transporter; Rib1-5/7p, enzymes involved in Rf* de novo* biosynthesis; Rf_*T*_, mitochondrial riboflavin transporter; Fmn1p, riboflavin kinase; mtFADS, mitochondrial FAD synthase; Flx1p, mitochondrial FAD exporter; I, FAD pyrophosphatase; Sdh1p, succinate dehydrogenase flavoprotein subunit; Sdh5p, protein required for Sdh1p flavinylation; Sdh2/3/4p, other subunits of succinate dehydrogenase complex; Tmp62p/Sdh6p, factors required for SDH complex assembly; TCA cycle, tricarboxylic acid cycle; TOM complex/TIM complex, proteins involved in mitochondrial protein import; Dic1p, mitochondrial dicarboxylic acid carrier; PDH, prolyl hydroxylase; JmjC, JmjC-domain-containing demethylases, Rox1p, heme-dependent repressor of hypoxic genes; Msn2/4p, transcriptional factors activated in stress conditions.

In [19] we demonstrated that the early-onset change in apo-Sdh1p content observed in the* flx1Δ* strain appeared consistent with a posttranscriptional control exerted by Flx1p, as depicted in Figure 6. Thus, an inefficient translation of SDH1-mRNA is expected in* flx1Δ* strain due to the posttranscriptional control [19], even when putative mRNA levels may change in response to cell stress and/or aging. In this pathway the transcription factors Msn2/4p and Rox1p could play a crucial role.

Moreover, scheme in Figure 6 outlines how* FLX1* deletion, causing a change in expression level of Sdh1p, could activate a sort of retrograde cross-talk directed to nucleus. In our hypothesis besides ROS increase, a key molecule mediating nucleus-mitochondrion cross-talk should be the TCA cycle intermediate succinate, whose amount is expected to increase when altering the activity of SDH. The increased amount of succinate in turn may alter the activity of the *α*-ketoglutarate- and Fe(II)-depending dioxygenases among which there are (i) the JmjC-domain-containing demethylases [36], which may be causative of epigenetic events at the basis of precocious aging (for an exhaustive review on this point see [49]), and (ii) the prolyl hydroxylase (PDH), which may mimic a hypoxia condition in the cell [50].

## 5. Conclusions

Here we prove that in* S. cerevisiae* deletion of the mitochondrial translocator* FLX1* results in H_2_O_2_ hypersensitivity and altered chronological lifespan, which is associated with ATP shortage and ROS unbalance in nonfermentable carbon source. We propose that this yeast phenotype is correlated to a reduced ability to maintain an appropriate level of succinate dehydrogenase flavoprotein subunit [19], which in turn can either derange epigenetic regulation or mimic a hypoxic condition. Thus,* flx1Δ* strain provides a useful model system for studying human aging and degenerative pathologic condition associated with alteration in flavin homeostasis, which can be restored by Rf treatment [51, 52].

## Figures and Tables

**Table 1:** Endogenous flavin content in spheroplasts and mitochondria.

Carbon source	Strain	Spheroplasts			SCM		
		FAD pmoli mg^ −1^	FMN pmoli·mg^−1^	FAD/FMN	FAD pmoli mg^ −1^	FMN pmoli·mg^−1^	FAD/FMN
*Glycerol *	WT	157 ± 7	153 ± 7	*1.1 *	160 ± 10°	30 ± 10°	*4.8 *
	*fl* *x*1Δ	126 ± 11	110 ± 10	*1.1 *	140 ± 30°	40 ± 10°	*4.5 *

*Galactose *	WT	263 ± 10	189 ± 8	1.4	538 ± 32	103 ± 7	5.2
	*fl* *x*1Δ	207 ± 8*	195 ± 8	1.1	306 ± 15*	67 ± 11*	4.8

Spheroplasts and mitochondria (SCM) were prepared from WT and *flx*1Δ cells grown in glycerol or galactose (2%) up to the exponential growth phase (5 h). FAD and FMN content was determined in neutralized perchloric acid extracts, as described in *Materials and Methods*. Riboflavin amount was not relevant, and thus its value has not been reported. The means (±SD) of the flavin endogenous content determined in three experiments performed with different preparations are reported. °Data published in (Bafunno et al., 2004) [26]; statistical evaluation was carried out according to Student's *t*-test (*P < 0.05).

**Table 2:** List of motifs localized in the 1000 nucleotides upstream region of SDH1 ORF and identified by enriched conservation among all Saccharomyces species genome using the “Yeast Comparative Genomics—Broad Institute” database.

Number	Motif	Number of ORFs	Binding factor	Function
2	RTTACCCGRM	865	Reb1	RNA polymerase I enhancer binding protein
**14**	**YCTATTGTT**	**561**	**Unknown**	/
26	DCGCGGGGH	285	Mig1	Involved in glucose repression
**29**	**hRCCCYTWDt**	**442**	**Msn2/4**	**Involved in stress conditions**
**38**	**CTCCCCTTAT**	**218**	**Msn2/4**	**Involved in stress conditions**
39	GCCCGG	152	Unknown	Filamentation
41	CTCSGCS	77	Unknown	/
47	TTTTnnnnnnnnnnnngGGGT	359	Unknown	/
57	CGGCnnMGnnnnnnnCGC	84	Gal4	Involved in galactose induction
61	GKBAGGGT	363	TBF1	Telobox-containing general regulatory factor
63	GGCSnnnnnGnnnCGCG	80	mbp1-like	Involved in regulation of cell cycle progression from G1 to S
70	CGCGnnnnnGGGS	156	Unknown	/

## Abbreviations

Rf: Riboflavin
RFK: Riboflavin kinase
FADS: FAD synthase
SCM: * Saccharomyces cerevisiae* mitochondria
WT: Wild-type
FUM: Fumarase
SDH: Succinate dehydrogenase
GR: Glutathione reductase
SOD: Superoxide dismutase
DCF-DA: 2′-7′-Dichlorofluorescin diacetate
TCA cycle: Tricarboxylic acid cycle.
